# The association between online gaming, social phobia, and depression: an internet survey

**DOI:** 10.1186/1471-244X-12-92

**Published:** 2012-07-28

**Authors:** Han-Ting Wei, Mu-Hong Chen, Po-Cheng Huang, Ya-Mei Bai

**Affiliations:** 1 Department of Psychiatry, Taipei Veterans General Hospital, Taipei, Taiwan; 2 Department of Rehabilitation, Taipei Hospital, Taipei, Taiwan; 3 Department of Psychiatry, Faculty of Medicine, National Yang-Ming University, No.201, Sec. 2, Shipai Rd, Taipei City, Beitou District, Taiwan

## Abstract

**Background:**

Online gaming technology has developed rapidly within the past decade, and its related problems have received increasing attention. However, there are few studies on the psychiatric symptoms associated with excessive use of online games. The aim of this study is to investigate the characteristics of online gamers, and the association between online gaming hours, social phobia, and depression using an internet survey.

**Methods:**

An online questionnaire was designed and posted on a popular online game websites, inviting the online gamers to participate the survey. The content of the questionnaire included demographic data, profiles of internet usage and online gaming, and self-rating scales of Depression and Somatic Symptoms Scale (DSSS), Social Phobia Inventory (SPIN), and Chen Internet Addiction Scale (CIAS).

**Results:**

A total of 722 online gamers with a mean age of 21.8 ± 4.9 years completed the online survey within one month. 601 (83.2%) participants were male, and 121 (16.8%) were female. The mean weekly online gaming time was 28.2 ± 19.7 hours, which positively associated with history of online gaming (r = 0.245, p < 0.001), total DSSS (r = 0.210, p < 0.001), SPIN (r = 0.150, p < 0.001), and CIAS (r = 0.290, p < 0.001) scores. The female players had a shorter history of online gaming (6.0 ± 3.1 vs. 7.2 ± 3.6 years, p = 0.001) and shorter weekly online gaming hours (23.2 ± 17.0 vs. 29.2 ± 20.2 hours, p = 0.002), but had higher DSSS (13.0 ± 9.3 vs. 10.9 ± 9.7, p = 0.032) and SPIN (22.8 ± 14.3 vs. 19.6 ± 13.5, p = 0.019) scores than the male players. The linear regression model showed that higher DSSS scores were associated with female gender, higher SPIN scores, higher CIAS scores, and longer weekly online gaming hours, with controlling for age and years of education.

**Conclusion:**

The online gamers with longer weekly gaming hours tended to have a longer history of online gaming, and more severe depressive, social phobic, and internet addiction symptoms. Female online gamers had fewer weekly online gaming hours and a shorter previous online gaming history, but tended to have more severe somatic, pain, and social phobic symptoms. The predictors for depression were higher social phobic symptom, higher internet addiction symptoms, longer online gaming hours, and female gender.

## Background

In recent years, internet addiction has been regarded as an increasingly significant public health issue. A range of studies have demonstrated that a high prevalence of internet addiction in adolescents and young adult population is associated with evident psychiatric problems and serious functional impairment [1-4]. In Greek, Siomos et al’s study revealed that 8.2% of 2200 adolescent students aged between 12 to 18 had meet the criteria of the Diagnostic Questionnaire for Internet Addiction [4]. In US, Christakis et al. assessed 307 college students at two US universities using the Internet Addiction Test and the Patient Health Questionnaire, noting that 4% of students scored in the problematic or addicted range, and identifying a significant association between problematic internet usage and moderate to severe depression [2]. Among 50 adult patients with internet addiction, Bernadi et al. observed that 14% had a diagnosis of attention deficit and hyperactivity disorder, 15% generalized anxiety disorder, 15% social anxiety disorder, and 7% dysthymia [5]. In Taiwan, Tsai et al. identified 17.9% of university students meet the criteria of internet addiction group using the Chinese Internet Addiction Scale-Revision (CIAS-R), and the risk factors included male gender, habit of skipping breakfast, mental health morbidity, deficient social support, and neurotic personality traits [6].

The technology of online gaming has rapidly developed within the past decade, with online games becoming one of the major daily entertainments for millions of people. Prior research identified that, among the internet activities, online gaming plays an important role on internet addiction, associating with poorer prognosis and more severe social impairments [7]. Griffiths et al. reported that 80% of online gamers sacrificed at least one element of their lives, such as sleep, work, education, and socializing with friends, family, and partners, to play online games. The younger the players, the longer the time they dedicated to playing online games, associating with the further functional impairment [8]. There are four main attractions to online gaming. First, the original game design: the soundtrack, frames, background story, and the complexity of the gaming elements. Second, the role playing achievements: the online gamers may experience new virtual roles, gaining satisfaction from building up characters within levels, accumulating online resources, experiencing online adventures, and receiving online rewards. Third, online social interactions: the gamers may form virtual relationships, gaining online friends, lovers, virtual business, and conducting other types of online activities. Fourth, psychological needs and motivations: online games provide players with outlets for unsatisfying needs and motivations in the real life; within the online game world, players can acquire those they are seeking in the real life, most of the times in an easier way, which motivates them to keep on gaming [9]. The craving for online gaming and substance dependence may share similar neurobiological mechanisms, thus inducing analogous behavioral effects such as excessive usage, severe withdrawal symptoms, tolerance, and negative repercussions [10]. Although there are many previous related studies on internet addiction, but few studies specifically focus on the online gaming and associated psychiatric problem. The aim of this study was, therefore, to investigate the characteristics of online gamers and the association between online gaming hours, social phobia, and depression, using an internet survey.

## Methods

### Participants and procedure

An online questionnaire was designed and posted from August 1 to August 31, 2010 on the most popular online game websites in Taiwan (PTT BBS and 3 C gamer) to invite the online gamers participate the survey. The participant had to fill out the online informed consent; which explained the answers will be analyzed only for the research purpose, and there is no way to link the data to their true identity. After filling out the online informed consent, the participants would answer an online questionnaire. All participants were assigned a random number in the survey by the server, and they didn't have to fill out the name. Experiments were conducted in accordance with the Declaration of Helsinki and approved by the Institutional Review Board of Taipei Veterans General Hospital. Online agreed informed consent was obtained from all the subjects with adequate understanding of the study.

### Questionnaire design

The questionnaire composed of five sections: 1) Demographic information, including age, gender, education years, etc.; 2) Profiles of internet usage and online gaming, including weekday and weekend online gaming hours and total internet hours, history of past online gaming years, etc.; 3) Depression and Somatic Symptoms Scale (DSSS); 4) Social Phobia Inventory (SPIN); 5) Chen's Internet Addiction Scale (CIAS).

### Depression and Somatic Symptoms Scale (DSSS)

It is a 22-item self-administered rating scale, including three subscales as the Depression Subscale (DS), Pain Subscale (PS), and Somatic Subscale (SS). The DS had 12 items, including three vegetative symptoms and fatigue, and the SS had 10 items, including five pain items, which comprised the 5-items pain subscale (PS). Each item is rated with 0-3 score: 0 (not at all); 1 (mild); 2 (moderate); 3 (severe). The range of the sum score is thus 0–66. The scale had good validity and reliability, and higher the scores demonstrate heavier the symptoms [11].

### Social Phobia Inventory (SPIN)

It is a 17-item self-administered rating scale for evaluating the severity of social phobic symptoms, including three components: Fear in social situations (6 items), Avoidance of performance or social situations (7 items), and physiological discomfort in social situations (4 items). Participants were asked to score the distress of each symptoms according to the frequency during the past week: 0 (not at all); 1 (a little bit); 2 (somewhat); 3 (very much); or 4 (extremely). The scale had good validity and reliability, and higher the scores demonstrate heavier the symptoms [12].

### Chen's Internet Addiction Scale (CIAS)

It is a 26-item self-administered rating scale for internet addiction, including 5 dimensions: Compulsive use, Withdrawal, Tolerance, Problems of interpersonal relationships, and Health and Time management. The total scores of the CIAS ranged from 26 to 104. Higher CIAS scores indicated increased severity of Internet addiction. The scale had good validity and reliability [13,14].

Statistical analysis. Statistical analysis was performed using Statistical Package for Social Science (SPSS) version 17 software (SPSS Inc, Chicago, IL). Analysis of Variance (ANOVA) and Pearson chi-square test were applied to compare the continuous (age, years of education, years of online gaming, DSSS score, DSSS-DS/PS/SS subscale scores, CIAS score, SPIN score) and categorical (gender) variables among the four groups of online gamers according to weekly online gaming hours. Bonferroni post-hoc analysis was performed to investigate the significance of CIAS scores, DSSS scores, DSSS-DS/PS/SS scores, and SPIN scores among four groups of online gamers. Correlation test was also performed to investigate the correlation among weekly online gaming hours, DSSS scores, SPIN scores, and CIAS scores. Effects of gender on psychiatric symptoms and patterns of online gaming among the two genders were also analyzed. The linear regression model was performed to determine the predictors of addictive symptoms (CIAS score) and depressive symptoms in the online gamers. All statistics were two-tailed and a p value of <0.05 was considered significant.

## Results

**Figure 1:**
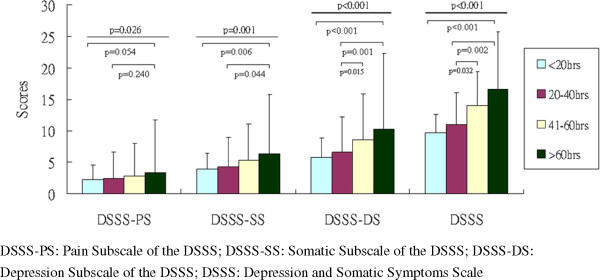
Weekly online gaming hours and association with DSSS-PS, DSSS-SS, DSSS-DS, and DSSS.

**Figure 2:**
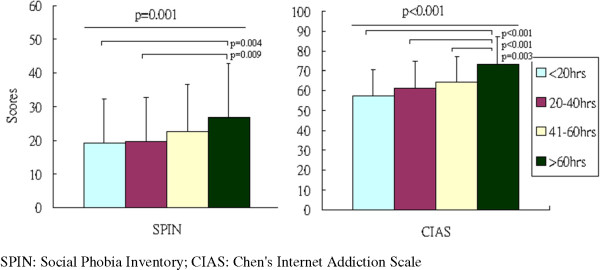
Weekly online gaming hours and association with SPIN and CIAS.

A total of 722 online gamers, with a mean age of 21.8 ± 4.9 years, completed the online survey within one month. 601 (83.2%) participants were males and 121 (16.8%) were females. Regarding the effects of working days and holidays on online gaming playing in Wenzel et al.’s study that online gamers were divided into four groups by daily online game time (less than 1 hour daily, 1-2 hours daily, 2-4 hours daily, and > 4 hours daily) [15], the weekly online gaming hours was deemed as the observed parameter in our study. The mean weekly online gaming time was 28.2 ± 19.7 hours. The online gamers were divided into four groups according to their weekly online gaming time: less than 20 hours (n = 297, 41%), 20 to 40 hours (n = 270, 37%), 40 to 60 hours (n = 112, 16%), and more than 60 hours (n = 43, 6.0%). Among the four groups, male gender, and longer history of online gaming significantly associated with longer weekly online gaming hours. There were no significant differences in age and years of education between the groups (Table 1). Figure 1 and 2 depicted significant dose-dependent effects of weekly online gaming hours on DSSS scores and its subscale scores (DSSS-PS scores, DSSS-SS scores, DSSS-DS scores), CIAS scores, and SPIN scores among four groups of online gamers. Furthermore, according to the correlation test, the weekly online gaming hours positively correlated with history of online gaming (r = 0.245, p < 0.001), total DSSS (r = 0.210, p < 0.001), DSSS-DS (r = 0.220, p < 0.001), DSSS-SS (r = 0.156, p < 0.001), DSSS-PS (r = 0.131, p < 0.001), SPIN (r = 0.150, p < 0.001), and CIAS (r = 0.290, p < 0.001) scores.

**Table 1:** Online gaming hours and association with gender, age, education, and history of online gaming

	**Weekly online gaming hours**					
	**<20**	**20 ~ 40**	**41 ~ 60**	**> 60**	**F value**	** *P* value**
	**(n = 297)**	**(n = 270)**	**(n = 112)**	**(n = 43)**		
Male, n	240 (80.8%)	221 (81.9%)	103 (92.0%)	37 (86.0%)	2.677	0.046 *
Age, yrs	21.5 (5.0)	22.1 (5.1)	22.3 (4.1)	21.2 (4.6)	1.577	0.194
Education, yrs	15.0 (2.7)	15.0 (2.4)	14.9 (2.5)	14.4 (2.6)	1.028	0.379
History of online gaming, yrs	6.2 (3.8)	7.2 (3.1)	8.5 (3.2)	8.0 (3.8)	14.374	<0.001 **

*: p < 0.05, **: p < 0.001.

There were significant differences in online gaming use between the genders. The female players had a shorter history of online gaming (6.0 ± 3.1 vs. 7.2 ± 3.6 years, p = 0.001) and shorter weekly online gaming hours (23.2 ± 17.0 vs. 29.2 ± 20.2 hours, p = 0.002), but had higher total DSSS (13.0 ± 9.3 vs. 10.9 ± 9.7, p = 0.032), DSSS-SS (5.56 vs. 4.22, p = 0.002), DSSS-PS (3.23 vs. 2.37, p = 0.001), and SPIN (22.8 ± 14.3 vs. 19.6 ± 13.5, p = 0.019) scores than the male players (Table 2).

**Table 2:** Gender differences in age, education, history of online gaming, weekly online gaming hours, DSSS, DSSS-DS, DSSS-SS, DSSS-PS, SPIN, and CIAS

	**Male**	**Female**	**F value**	**P value**
	**(N = 601)**	**(N = 121)**		
Age, yrs	21.7 (4.8)	22.6 (5.2)	3.26	0.072
Education, yrs	15.0 (2.5)	15.0 (2.6)	0.08	0.782
History of online gaming, yrs	7.2 (3.6)	6.0 (3.1)	11.58	0.001 *
Weekly online gaming hours	29.2 (20.0)	23.2 (17.0)	9.57	0.002 *
DSSS	10.9 (9.7)	13.0 (9.3)	4.62	0.032 *
DSSS-DS	6.7 (6.0)	7.7 (5.6)	1.16	0.283
DSSS-SS	4.2 (4.6)	5.7 (4.8)	9.70	0.002 *
DSSS-PS	2.4 (2.5)	3.2 (2.7)	11.40	0.001 *
SPIN	19.6 (13.5)	22.8 (14.3)	5.50	0.019 *
CIAS	61.1 (14.6)	60.1 (14.0)	0.52	0.473.

DSSS: Depression and Somatic Symptoms Scale, and its subscales of Depression Subscale (DSSS-DS), Somatic Subscale (DSSS-SS), and Pain Subscale (DSSS-PS); SPIN: Social Phobia Inventory; CIAS: Chen Internet Addiction Scale; *: p < 0.05.

Regarding depressive symptoms, the multivariate linear regression model (F = 44.766, R^2^ = 0.305, p < 0.001) showed higher SPIN scores (β = 0.310, p < 0.001), higher CIAS score (β = 0.308, p < 0.001), longer weekly online gaming hours (β = 0.091, p = 0.013), and female gender (β = 0.063, p = 0.026) as significant predictors of high DSSS scores after controlling for age and years of education (Table 3). Furthermore, regarding addictive symptoms (CIAS score), the multivariate linear model (F = 71.769, R^2^ = 0.282, p < 0.001) demonstrated higher DSSS scores (β = 0.312, p < 0.001), higher SPIN score (β = 0.224, p < 0.001), longer weekly online gaming hours (β = 0.175, p < 0.001), and longer history of online gaming (β = 0.066, p = 0.043) as significant predictors of high CIAS scores after controlling age, gender, and years of education (Table 4).

**Table 3:** Linear regression model for prediction of DSSS

	**Effect on Slope**		
	β	**t**	**Sig.**
SPIN	0.310	9.061	<0.001 **
CIAS	0.308	8.749	<0.001 **
Weekly online gaming hours	0.091	2.685	0.007 *
Female gender	0.063	1.976	0.049 *
Age	0.056	1.504	0.133
History of online gaming	-0.036	-1.074	0.283
Education	0.020	0.549	0.583

Linear regression model: F = 44.766, R^2^ = 0.305, Adjusted R^2^ = 0.298, p < 0.001; Dependent variable: DSSS (Depression and Somatic Symptoms Scale); Predictors: SPIN (Social Phobia Inventory), CIAS (Chen Internet Addiction Scale), Weekly online gaming hours, Female gender, Age, History of online gaming, Education; *: p < 0.05, **: p < 0.001.

**Table 4:** Linear regression model for prediction of CIAS

	**Effect on Slope**		
	**β**	**t**	**Sig.**
DSSS	0.469	8.730	<0.001 **
SPIN	0.238	6.332	<0.001 **
Weekly online gaming hours	0.129	5.263	<0.001 *
History of online gaming	0.272	2.029	0.043 *
Age	-0.009	-0.277	0.782
Female gender	-0.045	-1.385	0.167
Education	0.025	0.767	0.443

Linear regression model: F = 71.769, R^2^ = 0.286, Adjusted R^2^ = 0.282, p < 0.001; Dependent variable: CIAS (Chen Internet Addiction Scale); Predictors: SPIN (Social Phobia Inventory), DSSS (Depression and Somatic Symptoms Scale), Weekly online gaming hours, Female gender, Age, History of online gaming, Education; *: p < 0.05, **: p < 0.001.

## Discussion

Our results showed a positive correlation between weekly online gaming hours and internet addiction symptom. The results were consistent with Ko et al.'s study showing a positive correlation between total online gaming hours and total CIAS score, indicating that excessive use of online games resulted in higher risk of internet addiction, leading to more functional impairment, including failure to fulfill obligations at work, school, and home, and decreased participation in social or recreational activities [13].

Our results also showed a positive correlation between online gaming hours and depressive symptom (DSSS-Depressive Subscale), somatic symptom (DSSS-Somatic Subscale), and Pain symptom (DSSS-Pain Subscale). The association of depressive symptoms was consistent with the findings of Schimit et al.’s study that subjects with online video game dependency spent longer hours per week playing games, had higher scores for loneness or isolation, higher scores for depression, lower scores for social belonging in real life, lower scores for self-esteem, and reduced ability to cope with emotional problems compared with those without dependency [16]. In Achab et al.’s study comparing the characteristics of addict vs non-addict online gamers, these addicted gamers self-reported significantly higher rates (3 times more) of irritability, daytime sleepiness, sleep deprivation due to play, low mood and emotional changes since online gaming onset [17]. Furthermore, self-reported negative consequences of computer game playing increased strongly with average daily playing time and the prevalence of sleeping problems, depression, suicide ideations, anxiety, and obsessions/ compulsions increased with increasing playing time in Wenzel et al.’s study [15]. Previously, investigators have proposed that subjects with depression use the internet excessively as a means of self-medicating, and that internet addiction itself could also cause depressive symptoms. Internet addiction and depression may share similar risk factors, such as environment, genes, education, or stress-coping skills, and each might serve to exacerbate the severity of the other [18]. In terms of personality traits, previous studies identified that individuals with online game addiction, especially the Massive Multiplayer Online Role Playing Games (MMORPG), had more aggressive and narcissistic tendencies, less self-control, fewer real world achievements, and lower self-esteem than normal individuals [19]. However, further investigation is needed to elucidate the common mechanisms underlying internet addiction and depression.

For the association of online gaming hours and somatic/pain symptoms, it might be explained that excessive game-playing leaded to muscle soreness, dry eyes, sleep deprivation, inadequate exercising, and even changes in dietary habits [20]. However, previous studies had shown the patients with depression had more somatic and pain symptoms. Half the depressed patients reported multiple unexplained somatic symptoms, and denied psychological symptoms of depression on direct questioning [21]. Some previous studies have suggested that patients in non-Western countries are more likely to report somatic symptoms than are patients in Western countries. The presence of any physical symptom increased the likelihood of a diagnosis of a mood or anxiety disorder by at least twofold to three-fold [22]. These online gamers might not identify their depression, but feel many somatic symptoms such as headache, chest tightness, and muscle pain to make them can't focus on school or work, and just spent much time on online game. For the clinical implication, the online gamer who complain many somatic and pain symptoms, we should pay attention to the possibility of depression.

Our results also demonstrated a positive correlation between online gaming hours and social anxiety symptoms by SPIN score. These results suggest that players who suffer from social phobic symptoms are more likely to indulge in the virtual reality provided by online games to avoid real life face to face social distress. Previous studies had shown the individuals with internet addiction had psychopathological characteristics of low self-esteem, low self-perception, and low confidence, but this social detachment in internet could result in further interpersonal frustrations in players’ real lives [23-25]. Achab et al. demonstrated online gamers with positive dependence Adapted Scale had more social, financial, marital, family, and/or professional difficulties since they started online gaming [17]. These findings highlighted the importance of identifying the problem of social anxiety/phobia when treating excessively using online gamers.

Another interesting finding is the gender difference. In our present study, 121 (16.8%) of the participants were female, with similar ages, years of education, and CIAS scores to the male online gamers. Females form a smaller proportion of the online gaming population. They also had shorter histories of online gaming and shorter weekly online gaming hours, but had more severe somatic, pain, and social phobic symptoms than the male players. The regression model also indicated the female gender is a predictor of depression according to DSSS score. Actually the gender difference has been identified in previous studies of substance addiction. Tuchman et al. reported gender differences in motivations for substance abuse, with females more likely to use illicit drugs for self-medication of depression or as a means of coping with stressful life events [26]. Women with substance-use problems are with more familial circumstances such as domestic violence, over-responsibility and divorce as high impact factors that lead to drug abuse [27]. Among 425 undergraduate students with problematic internet use, Hetzel-Riggin et al. reported that depression, keeping to oneself, and decreased tension increased problematic internet use in female online gamers [24]. In general, the majority of online gamers were males, these female online gamer had shorter histories of online gaming and shorter weekly online gaming hours, but had more severe somatic, pain, and social phobic symptoms than the male players. The results indicated these female players tend to engage in online games as a means of coping with depression, somatic symptoms, pain symptoms, and social anxiety. From the clinical point of view, the female online gamer might be with higher risk of depression.

To our best knowledge, this is the first study to investigate excessive online game hours and its association with depressive, social phobic, and internet addiction symptoms. However, there were some limitations. First, the enlisting of study subjects via invitation from online gaming websites introduces selection bias, thus the validity of their responses cannot be ensured. Second, the study is a descriptive, cross-sectional study. A prospective study would have represented a more meaningful means of evaluating the causal relationship between long hours spent playing online games and depression, social phobia, and internet addiction. Third, diagnoses of internet addiction, depression and social phobia could not be confirmed through self-completed questionnaires. Further investigation by face-to-face interview is needed to validate the findings. Fourth, the positive correlation between time spent on gaming and the internet addiction scale may be different for the subgroup of people with highly skilled hobbies or professions. In our survey, we didn’t identify this factor, and it deserves further research.

## Conclusion

In conclusion, in the study population, online gamers who played excessively had higher incidence of comorbidities including internet addiction, depression, and social phobia. Depressive symptoms increased in severity with longer weekly online gaming hours, female gender, and severity of social phobia symptoms. These findings could prove useful when devising future strategies for prevention and intervention of problematic online gaming habits.

## Competing interests

The authors declare that they have no competing interests.

## Author’s contribution

Wei, Han-Ting carried out the major study design and drafted the manuscript. Bai, Ya-Mei carried out the design and coordination of the team, as well as the interpretation of the data. Chen, Mu-Hong carried out the statistical analysis and graph designs. Haung, Po-Cheng carried out the questionnaire designs and collection. All authors have approved the final manuscript.

## Pre-publication history

The pre-publication history for this paper can be accessed here:

http://www.biomedcentral.com/1471-244X/12/92/prepub
